# Prognostic Role of Hemoglobin Combined With Geriatric Nutritional Risk Index in Patients With Vater Ampulla Carcinoma Undergoing Pancreaticoduodenectomy

**DOI:** 10.1002/cam4.70334

**Published:** 2024-10-14

**Authors:** Jieping Ying, Suwen Zhu, Yingchun Cheng, Bei Wang, Yueqin Wang

**Affiliations:** ^1^ Department of General Surgery, Yancheng Clinical Medical College of Jiangsu University The First people's Hospital of Yancheng Yancheng Jiangsu China; ^2^ Department of Medical Records, Yancheng Clinical Medical College of Jiangsu University The First people's Hospital of Yancheng Yancheng Jiangsu China; ^3^ Nursing Department, Yancheng Clinical Medical College of Jiangsu University The First people's Hospital of Yancheng Yancheng Jiangsu China

**Keywords:** geriatric nutritional risk index, hemoglobin, pancreaticoduodenectomy, Vater ampulla carcinoma

## Abstract

**Objectives:**

The purpose of this investigation was to assess the prognostic importance of the combination of preoperative hemoglobin (Hb) levels and Geriatric Nutritional Risk Index (GNRI) in forecasting postoperative survival outcomes for patients undergoing pancreaticoduodenectomy (PD) due to Vater ampulla carcinoma (VPC).

**Methods:**

The GNRI nutritional screening was conducted for all patients, and patient outcomes, including overall survival (OS), were subsequently monitored. An H‐ GNRI scoring system was established using the optimal critical values of 125.5 g/L for Hb and 91.72 for GNRI, as determined by X‐tile software. According to the H‐GNRI score, the patients were categorized into three groups, namely low H‐GNRI group (H‐GNRI score = 0, *n* = 47) with Hb < 125.5 g/L and GNRI < 91.72; medium H‐GNRI group (H‐GNRI score = 1, *n* = 77) with Hb < 125.5 g/L and GNRI ≥ 91.72, or Hb ≥ 125.5 g/L and GNR < 91.72; and high H‐GNRI group (H‐GNRI score = 2, *n* = 51) with Hb ≥ 125.5 g/L and GNRI ≥ 91.72. The Kaplan–Meier analysis and log‐rank tests were employed to evaluate the OS rate and compare survival disparities among various groups. Additionally, both univariate and multivariate analyses were conducted utilizing the Cox regression model, with *p* < 0.05 considered statistically significant. Finally, to evaluate the predictive effectiveness of Hb, GNRI, and H‐GNRI, a receiver operating characteristic (ROC) curve was constructed to compare the area under curve (AUC) values.

**Results:**

The OS rate was higher in patients with high Hb levels (≥ 125.5 g/L) compared to those with low Hb levels (< 125.5 g/L). Likewise, patients in the high GNRI group (≥ 91.72) exhibited significantly superior OS compared to those in the low GNRI group (< 91.72). Compared with both the medium and low H‐GNRI groups, the high H‐GNRI group demonstrated a notably higher OS rate. The T stage (HR = 2.523, 95% CI: 1.694–3.757, *p* < 0.001), N stage (HR = 2.018, 95% CI: 1.255–3.246, *p* = 0.004), and the H‐GNRI score (H‐GNRI score of 2 used as the baseline; H‐GNRI score of 0: HR = 2.569, 95% CI: 1.499–4.402, *p* < 0.001; H‐GNRI score of 1: HR = 1.835, 95% CI: 1.118–3.014, *p* = 0.016), after adjusting for gender, were determined to be independent significant predictors affecting the OS of patients with VPC. The AUC of H‐GNRI was 0.677, exceeding that of Hb levels (0.631) and GNRI (0.615).

**Conclusions:**

The combination of preoperative Hb levels and GNRI demonstrates superior predictive efficacy for VPC patients undergoing PD, compared with either Hb levels or GNRI score alone. Therefore, the H‐GNRI score can be utilized to promptly identify high‐risk patients, establish comprehensive nutritional pre‐rehabilitation plans through interdisciplinary collaboration, and inform decisions regarding additional adjunctive therapies.

**Abbreviations**
AUC: area under curve
CONUT: Controlling Nutritional Status Score
GNRI: Geriatric Nutritional Risk Index
Hb: hemoglobin
OS: overall survival
PD: pancreaticoduodenectomy
PNI: Prognostic Nutritional Index
ROC: receiver operating characteristic
VPC: Vater ampulla carcinoma

## 1 Introduction

Vater ampulla carcinoma (VPC) refers to tumors that occur near the ampulla of Vater, at the distal end of the bile duct and in the vicinity of the duodenal papilla. It predominantly comprises cancers of the ampulla, distal bile duct, duodenum, and pancreatic head, and it frequently occurs in individuals aged from 40 to 70 years [1]. In clinical practice, pancreaticoduodenectomy (PD) is mainly used to treat VPC. This surgical procedure is one of the primary methods for achieving radical treatment of the disease [2]. In recent years, due to the continuous refinement of minimally invasive techniques and significant optimization of perioperative management strategies, the mortality rate after PD has decreased. However, despite a significant increase in surgical safety, the occurrence rate of postoperative complications following PD remains high, spanning from 30% to 50% [3], which greatly affects the patient's prognosis [4, 5]. Therefore, the evaluation of postoperative recovery and survival prognosis of patients undergoing PD is still a significant clinical issue requiring resolution. It is of great significance to find effective biomarkers and predictive factors to more accurately evaluate the survival risk of patients after surgery.

Hemoglobin (Hb), a prevalent biological marker, mirrors the body's oxygen‐carrying capacity and overall health status, and it exhibits a strong correlation with the survival prognosis of cancer patients. Previous studies have confirmed that preoperative Hb levels significantly affect the prognosis of patients with solid tumors, such as lung cancer, colorectal cancer, cervical cancer, and bile duct cancer [6, 7]. The Geriatric Nutritional Risk Index (GNRI) represents an uncomplicated and objective nutritional assessment instrument that is determined based on serum albumin concentrations, the individual's present body weight, and the person's ideal body weight [8]. The utilization of GNRI as a prognostic marker has been verified in individuals diagnosed with gastric cancer, hepatocellular carcinoma, pancreatic cancer, and head and neck malignancies [9, 10, 11]. However, to our understanding, there is currently a lack of research investigating the prognostic implications of preoperative GNRI for patients undergoing PD surgery for VPC. Moreover, it remains uncertain whether the combination of Hb levels and GNRI influences the prognosis of patients with VPC.

Hence, this study was conducted to explore the correlation between preoperative Hb levels, in conjunction with GNRI, and the long‐term survival outcome of patients undergoing PD for VPC.

## 2 Materials and Methods

### 2.1 Patients

A total of 175 patients undergoing PD for VPC between January 2018 and December 2021 were included, and their pathological data were analyzed retrospectively. The inclusion criteria were as follows: (1) age ≥ 18 years, with complete clinical case data and follow‐up information; (2) histopathologically confirmed diagnosis of VPC; (3) radical PD. The exclusion criteria were as follows: (1) incomplete clinical case data or follow‐up information; (2) preoperative chemotherapy, radiotherapy, or other antitumor therapies; (3) presence of concurrent malignant tumors; (4) preoperative evidence of infection or inflammatory diseases; (5) coexistence of severe cardiovascular, cerebrovascular, hepatic, or renal diseases; (6) recurrent VPC; (7) presence of comorbid mental or psychological disorders. Pancreatic surgeons with a high level of expertise performed the surgical procedures. Informed consent was obtained from all study participants prior to their involvement. Both inpatient and outpatient medical records were utilized to gather extensive clinical data. This study was granted approval by the Ethics Committee of the First People's Hospital of Yancheng in the year 2021 (approval number: 2024‐k‐171) and adhered to the revised Declaration of Helsinki (2013).

### 2.2 Calculation of GNRI

The following formula was utilized to calculate the GNRI [12]: GNRI = [14.89 × serum albumin level (g/dL)] + [41.7 × actual body weight/ideal body weight]. In cases where the preoperative weight exceeded the calculated ideal body weight, we set the ratio of the current weight to the ideal weight at 1 [8]. Pre‐surgery data on serum albumin and actual body weight were collected for all patients upon admission.

### 2.3 Gathering of Clinical and Laboratory Data

The preoperative parameters encompassed various clinical records, including demographic variables (such as gender and age), nutrition‐related indicators (such as preoperative height, weight, and body mass index; preoperative hemoglobin levels; and serum albumin levels), and clinicopathological factors (such as tumor size, tumor site, degree of differentiation, T stage, N stage, and clinical stage). These parameters were extracted from the patients' medical records. The pathological stages were determined in accordance with the TNM classification system (8th edition) [13]. The 8th edition of the UICC TNM Classification, published in 2016, offers enhanced standards for staging cancer, which is vital for predicting outcomes and informing treatment strategies. The TNM system classifies tumors by the extent of the primary tumor, lymph node involvement, and presence of metastasis, serving as a global standard to guide clinical decisions and assess patient prognosis.

### 2.4 H‐GNRI Scoring System

Using the X‐tile software, we determined the optimal threshold values for preoperative Hb levels and GNRI. The H‐GNRI scoring system was established based on these identified optimal thresholds. By combining the stratified values of both parameters, the patients were categorized into three groups for H‐GNRI scoring, namely, the H‐GNRI score of 2 was assigned when both Hb levels and GNRI were high; the H‐GNRI score of 1 was assigned when only one of the parameters was high, while the other was low; and the H‐GNRI score of 0 was assigned when both markers were low. This approach integrated the assessment of Hb levels and nutritional status to comprehensively evaluate the patient's health profile.

### 2.5 Follow‐Up Study

Every patient underwent standard blood tests before surgery, which encompassed evaluations of serum albumin and tumor markers, and a comprehensive physical assessment that comprised measurements such as weight and height. In the initial 2‐year period, follow‐up assessments were conducted at 3‐month intervals. This was followed by evaluations every 6 months during the third year, and subsequently, annual check‐ups were carried out. The overall survival (OS) of patients was ascertained via outpatient visits or telephonic interviews, calculating the duration from the surgical procedure until either demise or the latest follow‐up visit. Follow‐up data were available until December 31, 2023, or until patient's demise.

### 2.6 Statistical Analysis

The SPSS 26.0 software and R software 4.0.3 were employed to perform the statistical analysis, while X‐tile software was used for determining the best truncation values of Hb levels and GNRI. The Pearson's chi‐square test was utilized to evaluate the association between H‐GNRI score and clinicopathological factors, whereas the Kaplan–Meier method and the log‐rank test were implemented to determine the 3‐year OS rate and disparities in survival curves among different groups. Cox regression models were used for both univariate and multivariate analyses to identify the prognostic factors for OS. Variables exhibiting a significance level below 0.05 in the univariate analysis were incorporated into the multivariate analysis, wherein statistical significance was ascribed to a significance level of < 0.05. The ROC curve was constructed to assess the predictive efficacy of H‐GNRI score, Hb levels, and GNRI score for OS.

## 3 Results

### 3.1 Patients' Characteristics

Out of the 190 patients enrolled, a total of 175 were followed‐up with completely, whereas 15 patients were lost to follow‐up, resulting in a loss rate of 7.89%. The duration of follow‐up was 0.8–65 months, with a median duration of 45.8 months. The baseline data for the 175 patients are presented in Table 1. Among these patients, there were 104 males and 71 females. The age range of the participants was between 35 and 83 years, with a median age of 68 years. The causes of death were as follows: unknown etiology (37 cases), tumor progression (18 cases), malnutrition (9 cases), and a combination of tumor progression and malnutrition (30 cases). During the follow‐up period, 81 patients survived without any evidence of tumor recurrence. The 3‐year OS rate was 46.2% (81 out of 175 patients), with a median survival time of 25.4 months.

**TABLE 1:** Baseline characteristics of patients with periampullary carcinoma received PD (*n* = 175).

Characteristics	No. of patients	Proportion (%)
Gender		
Male	104	59.4
Female	71	40.6
Age		
< 60	61	34.9
≥ 60	114	65.1
Tumor location		
Ampulla	82	46.9
Pancreatic head	71	40.6
Distal bile duct	22	12.6
Differentiation		
Well	41	23.4
Moderate	54	30.9
Poor	55	31.4
Unknown	25	14.3
T classification		
T1‐T2	90	51.4
T3‐T4	85	48.6
N classification		
N0	148	84.6
N1‐Nx	27	15.4
BMI(kg/m^2^)		
< 24	113	64.6
≥ 24	62	35.4

Abbreviations: BMI, body mass index; N, lymph node; PD, pancreaticoduodenectomy; T, tumor.

### 3.2 Calculation of Hb and GNRI Cutoff Values by X‐Tile

**FIGURE 1:**
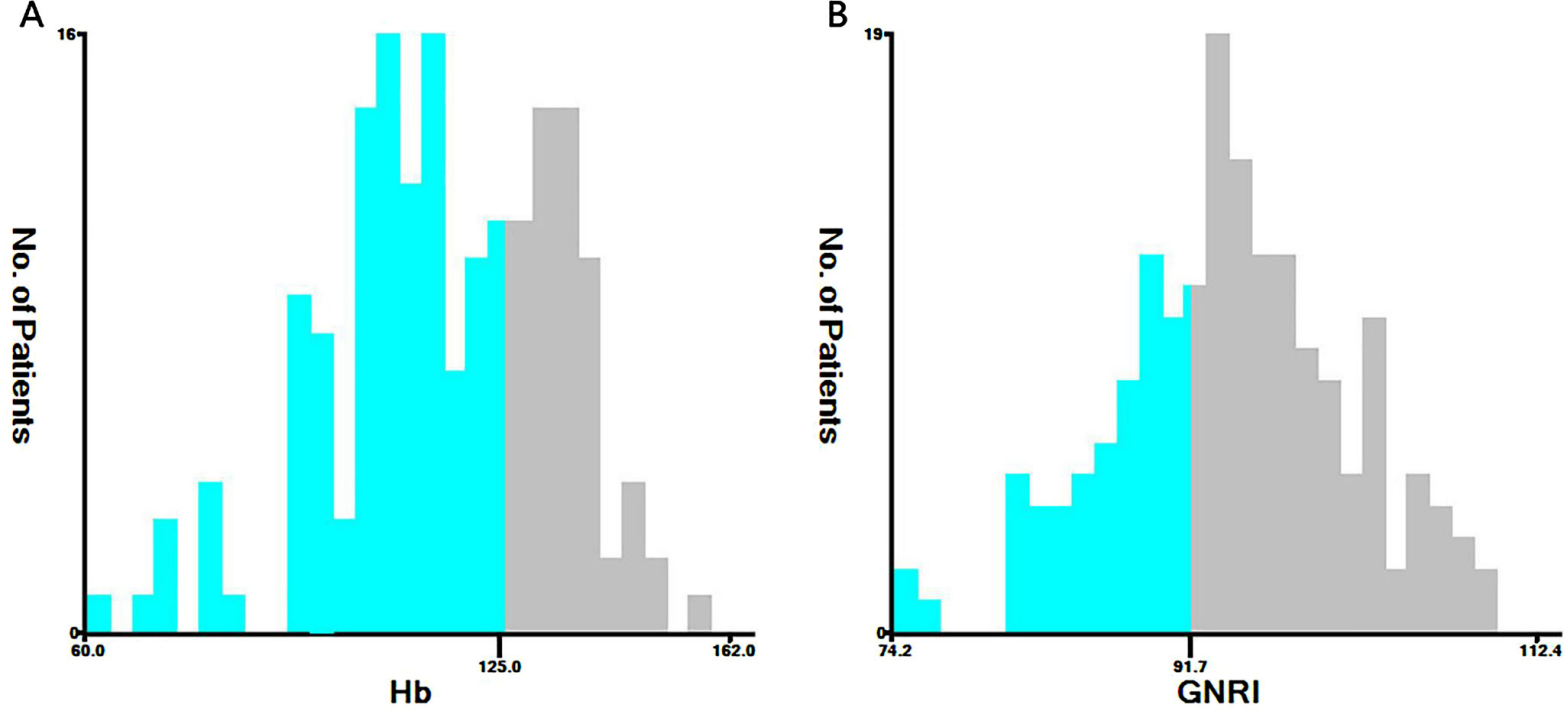
Optimal Hb and GNRI cutoff value: (A) Hb, (B) GNRI.

The optimal critical thresholds for Hb and GNRI were ascertained using X‐tile software analysis (Figure 1). The most appropriate cutoff values were 125.5 g/L for Hb (Figure 1A) and 91.72 for GNRI (Figure 1B). Based on these established thresholds, the patients were categorized into the following three groups: the low H‐GNRI group (H‐GNRI score = 0, *n* = 47), with Hb < 125.5 g/L and GNRI < 91.72; the medium H‐GNRI group (H‐GNRI score = 1, *n* = 77), with Hb < 125.5 g/L and GNRI ≥ 91.72, or Hb ≥ 125.5 g/L and GNRI < 91.72; and the high H‐GNRI group (H‐GNRI score = 2, *n* = 51), with Hb ≥ 125.5 g/L and GNRI ≥ 91.72. The relationship between the H‐GNRI score and clinicopathological characteristics of the patients was assessed (Table 2).

**TABLE 2:** Relationship between H‐GNRI score and clinicopathological characteristics (*n* = 175).

Factors	H‐GNRI score				
	0	1	2	*X* ^2^	*p*
Gender				23.023	< 0.001
Male	26	34	44		
Female	21	43	7		
Age				3.879	0.144
< 60	11	29	21		
≥ 60	36	48	30		
Tumor location				5.044	0.283
Ampulla	27	35	20		
Pancreatic head	13	34	24		
Distal bile duct	7	8	7		
Differentiation				8.648	0.194
Well	20	20	15		
Moderate	13	24	17		
Poor	12	17	12		
Unknown	2	16	7		
T classification				3.804	0.149
T1‐T2	21	46	23		
T3‐T4	26	31	28		
N classification				0.816	0.665
N0	41	63	44		
N1‐Nx	6	14	7		
BMI (kg/m2)				3.277	0.194
< 24	35	45	33		
≥ 24	12	32	18		

Abbreviations: BMI, body mass index; H‐GNRI score, hemoglobin and geriatric nutritional risk index score; N, lymph node; T, tumor.

### 3.3 OS Based on the Cutoff Value of Hb, GNRI, and H‐GNRI Score

**FIGURE 2:**
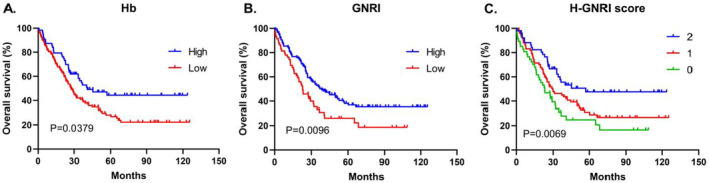
OS based on the cutoff value of Hb, GNRI and H‐ GNRI score: (A) Hb, (B) GNRI, (C) H‐GNRI.

The Kaplan–Meier analysis, coupled with the log‐rank test, indicated a significantly longer OS in the high Hb group than in the low Hb group (the OS rate: 47.6% vs. 28.6%; the median survival time: 40.6 months vs. 27.5 months; *p* = 0.0375; Figure 2A). Likewise, there was a notably longer OS in the high GNRI group compared with the low GNRI group (the OS rate: 39.6% vs. 27.1%; the median survival time: 38.63 months vs. 22.9 months; *p* = 0.0096; Figure 2B). Additionally, a substantial enhancement in the OS rate was detected in the high H‐GNRI group compared with both the medium and low H‐GNRI groups (the OS rate: 50.9% vs. 31.2% vs. 25.5%; the median survival time: 57.0 months vs. 29.8 months vs. 23.13 months; *p* = 0.0069; Figure 2C).

### 3.4 Cox Regression Analysis for OS

**FIGURE 3:**
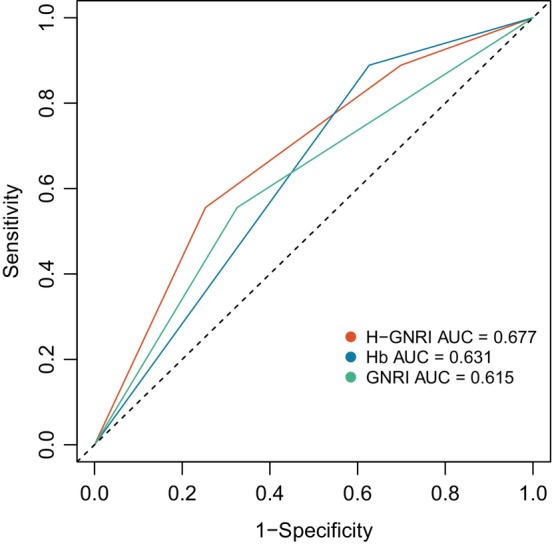
The ROC curve grouped by Hb, GNRI and H‐GNRI.

The univariate analysis revealed significant correlations between the OS and the degree of differentiation (*p* = 0.024), T stage (*p* < 0.001), N stage (*p* < 0.001), Hb levels (*p* = 0.019), GNRI (*p* = 0.011), and H‐GNRI score (*p* = 0.008). After adjusting for gender, the multivariate analysis demonstrated that T stage (HR = 2.523, 95% CI: 1.694–3.757, *p* < 0.001), N stage (HR = 2.018, 95% CI: 1.255–3.246, *p* = 0.004), and H‐GNRI score (H‐GNRI score of 2 used as the baseline; H‐GNRI score of 0: HR = 2.569, 95% CI: 1.499–4.402, *p* < 0.001; H‐GNRI score of 1: HR = 1.835, 95% CI: 1.118–3.014, *p* = 0.016) were significant prognostic factors for OS (Table 3). The AUC for the H‐GNRI was determined to be 0.677, surpassing the AUC values for Hb levels (0.631) and GNRI (0.615), as illustrated in Figure 3.

**TABLE 3:** Univariate and multivariate analyses for the prediction of overall survival in patients with periampullary carcinoma received PD (*n* = 175).

Factors	Univariate analysis			Multivariate analysis (gender‐adjusted)		
	HR	95% Cl	*p*	HR	95% Cl	*p*
Gender (male/female)	1.031	0.708–1.501	0.874	1.087	0.731–1.616	0.682
Age (≥ 60/< 60)	1.366	0.917–2.033	0.125			
Tumor location (baseline, distal bile duct)			0.174			
Ampulla	0.750	0.430–1.308	0.311			
Pancreatic head	1.093	0.629–1.899	0.752			
Differentiation (baseline, well)			0.024			
Moderate	0.703	0.421–1.172	0.177	—	—	—
Poor	1.211	0.748–1.959	0.436	—	—	—
Unknown	0.528	0.273–1.020	0.057	—	—	—
T classification (T3‐T4/T1‐T2)	2.696	1.832–3.968	< 0.001	2.523	1.694–3.757	< 0.001
N classification (N1‐Nx/N0)	2.431	1.534–3.853	< 0.001	2.018	1.255–3.246	0.004
BMI (< 24/≥ 24)	0.905	0.618–1.325	0.607			
H‐GNRI score (baseline, H‐GNRI score of 2)			0.008			
H‐GNRI score of 0	2.260	1.349–3.787	0.002	2.569	1.499–4.402	< 0.001
H‐GNRI score of 1	1.640	1.019–2.640	0.042	1.835	1.118–3.014	0.016

Abbreviations: BMI, body mass index; CI, confidence interval; H‐GNRI score, hemoglobin and geriatric nutritional risk index score; HR, hazard ratio; N, lymph node; PD, pancreaticoduodenectomy; T, tumor.

## 4 Discussion

The primary objective of this study was to develop a novel scoring system, known as the H‐GNRI, which integrates preoperative Hb levels with the GNRI. The aim was to evaluate its potential in predicting survival outcomes in patients with VPC undergoing PD and determine whether this composite indicator could offer a novel perspective on the patients' prognosis. We identified optimal cutoff values for Hb levels and GNRI at 125.5 g/L and 91.72, respectively, which were utilized for patient stratification purposes. Our findings revealed that high Hb levels and GNRI were associated with improved OS, highlighting the utility of these biomarkers in predicting patient outcomes. Specifically, the high H‐GNRI group demonstrated markedly better OS compared to the medium and low H‐GNRI groups, with the H‐GNRI score showing a superior predictive ability over Hb and GNRI levels alone. These results emphasize the importance of integrating both hematological and nutritional assessments in preoperative evaluations to better stratify patient risk and inform clinical decision‐making.

The role of Hb varies across different malignancies. Previous studies have consistently linked low preoperative Hb levels to unfavorable prognosis across various types of cancers [14, 15, 16, 17]. For example, Jatzko et al. [18] found that patients undergoing radical gastrectomy for gastric cancer with lower preoperative Hb levels had lower survival rates. A recent study has revealed that TNM stage III gastric cancer patients with preoperative anemia (defined as Hb levels < 120 g/L in males and < 110 g/L in females) had a 1.77‐fold higher risk of death compared with patients without anemia [19]. Similarly, previous studies have demonstrated a correlation between elevated preoperative Hb levels and a more favorable prognosis for patients with prostate cancer [20, 21]. This is in line with our study, as the patients in the low Hb group (< 125.5 g/L) exhibited a significantly shorter survival time than those in the high Hb group (≥ 125.5 g/L). The mechanism underlying the association between preoperative Hb levels and the prognosis of VPC patients, however, remains elusive. It is plausible that reduced Hb levels contribute to tumor hypoxia, thereby facilitating angiogenesis and augmenting cancer cell invasiveness. Consequently, these patients are more susceptible to metastasis, ultimately leading to an unfavorable prognosis [22].

In recent years, with the advancement of nutritional science, an increasing number of nutritional assessment systems have been developed, such as Controlling Nutritional Status Score (CONUT), Prognostic Nutritional Index (PNI), and GNRI. Each scoring system encompasses distinct indicators, evaluation methodologies, and simplicity. Given that the GNRI is widely recognized as a simple and accurate evaluation instrument for measuring serum albumin concentrations and body weight, it is often used to ascertain disease prognosis [23, 24, 25, 26, 27, 28]. Our findings indicated that the patients from the high GNRI group (≥ 91.72) exhibited a significantly more favorable prognosis than those from the low GNRI group (< 91.72). This is in line with previous research on survival outcomes in patients diagnosed with esophageal [29], gastric [9], and colorectal cancer [30]. Additionally, several studies conducted on patients diagnosed with pancreatic cancer support our findings [31, 32]. A recent retrospective study has yielded the same results, particularly in the case of patients diagnosed with stage II pancreatic cancer [33]. However, the findings from Itoh et al. [34] were incongruous with our results. Specifically, they conducted a comparative analysis of three scoring systems, namely PNI, CONUT, and GNRI, and revealed that solely PNI exhibited prognostic significance in terms of patient survival outcomes. The reason for this discrepancy may be attributed to the fact that their study utilized a large sample size, exclusively focused on elderly patients, and included two different surgical modalities, namely PD and distal pancreatectomy. The GNRI is correlated with personal serum albumin concentrations and body weight. Serum albumin serves not only as a marker for total protein content in the body but also as a significant biomarker reflecting nutritional status and prognosis [35, 36, 37, 38]. It is also acknowledged as an inflammatory marker. In the presence of inflammation, albumin escapes into extravascular tissues due to heightened vascular permeability. Proinflammatory mediators such as interleukin‐6, interleukin‐1, and tumor necrosis factor additionally impede albumin production [39]. Furthermore, chronic inflammation can augment tumor cell proliferation, metastasis, and immune evasion to facilitate tumorigenesis [40, 41]. These findings may elucidate the correlation between GNRI and patient outcomes.

The results of our study demonstrated that the patients with an H‐GNRI score of 2 had a significantly longer median survival time (57.0 months) than those with a score of 0 and 1 (23.1 months and 29.8 months, respectively). Additionally, the OS rate in this group was notably higher compared to the other two groups, with rates of 50.9%, 31.2%, and 25.5% respectively, and this difference was statistically significant (*p* = 0.0069), indicating a strong association between a high H‐GNRI score and improved prognosis. The results of the Cox regression model demonstrated that the H‐GNRI score, T stage, and N stage were the independent prognostic factors for OS in patients with VPC. As there was a significant difference in gender among the three H‐GNRI groups (*p* < 0.001), we adjusted for gender as a confounding factor. We found that the T stage (HR = 2.523, 95% CI: 1.694–3.757, *p* < 0.001), N stage (HR = 2.018, 95% CI: 1.255–3.246, *p* = 0.004), and H‐GNRI score (H‐GNRI score of 2 used as the baseline; H‐GNRI score of 0: HR = 2.569, 95% CI: 1.499–4.402, *p* < 0.001; H‐GNRI score of 1: HR = 1.835, 95% CI: 1.118–3.014, *p* = 0.016) were still independent prognostic factors for VPC patients. However, TNM staging can only be determined postoperatively, whereas Hb levels and GNRI can be assessed at any time before treatment initiation, providing a more convenient and expedited alternative to clinical TNM staging. Finally, through the construction of ROC curves, we observed that the H‐GNRI score exhibited significantly higher AUC values compared with Hb levels or GNRI alone. This finding confirms the superior accuracy of the H‐GNRI score in predicting OS compared with Hb levels or GNRI alone.

Our study has significant implications for clinical practice and, notably, for future research. First, the H‐GNRI score might be a supplementary indicator to inform clinical treatment decision‐making before PD in VPC patients with local invasion. The administration of treatments such as neoadjuvant chemotherapy may be necessary in high‐risk patients before surgery in order to reduce tumor size and enhance surgical success rates. Patients with a low H‐GNRI score exhibit reduced tolerance to neoadjuvant chemotherapy compared with those with a high H‐GNRI score, thereby potentially increasing their treatment‐related risks. Consequently, the incorporation of the H‐GNRI score can aid clinicians in adjusting treatment strategies. However, the clinical significance of the H‐GNRI score in preoperative adjuvant therapy for VPC patients remains unclear, necessitating further studies to ascertain its implications in the future. Second, we can utilize the H‐GNRI score as a foundation for preoperative evaluation and risk stratification, incorporating it into the preoperative nutritional risk assessment of patients. The patients with an H‐GNRI score of 0 are classified as being at a significantly elevated nutritional risk, necessitating the development of personalized nutritional pre‐rehabilitation plans that may encompass oral nutritional supplements, enteral nutrition support, or parenteral nutrition support. The expert group of the European Society for Parenteral and Enteral Nutrition has highlighted that preoperative malnutrition correlates with an elevated risk of postoperative complications, an extended duration of hospitalization, and diminished survival rates [42]. As early as 1980, Smale et al. [43] discovered that providing preoperative nutritional support to patients with cancer at high nutritional risk led to a twofold reduction in the occurrence of postoperative complications and a threefold decrease in severe sepsis and mortality. A recent meta‐analysis has demonstrated a significant reduction in the incidence of postoperative complications among colorectal cancer patients who received preoperative oral nutritional supplements [44]. Third, the H‐GNRI score can be used as a component of surgical risk assessment to aid surgeons in evaluating the patient's surgical tolerance and postoperative recovery process. For patients with a low H‐GNRI score, preoperative optimization measures such as blood transfusion or postponing surgery should be considered to enhance the patient's nutritional status and reduce systemic inflammation. The available evidence, however, suggests that perioperative transfusion therapy is associated with an increased risk of adverse events and postoperative complications [45]. Therefore, it remains imperative to explore alternative treatment options for managing anemia. In addition, the medical staff should conduct more stringent monitoring, intensive follow‐up, and possible interventions for such patients after surgery.

This study encountered several constraints. First, being a single‐center, retrospective cohort investigation with a comparatively modest sample size and potential biases related to region and population, the broader applicability of the results could be affected. Second, the focus of this study was solely on the collection and analysis of preoperative data, without incorporating other commonly utilized nutritional assessment tools and inflammatory indicators for comparative purposes. Third, we explored only the predictive role of the H‐GNRI score for OS, without considering other survival outcomes such as disease‐free survival and recurrence‐free survival. In the future, it is imperative to conduct multicenter, large‐sample prospective studies and incorporate additional blood parameters (such as systemic inflammation response index; neutrophil/lymphocyte ratio; monocyte/lymphocyte ratio; platelet/lymphocyte ratio) in order to comprehensively evaluate the merits and demerits of the H‐GNRI score and further elucidate its underlying predictive mechanism.

## 5 Conclusions

The combined assessment of preoperative Hb levels and GNRI may provide valuable prognostic information for patients with VPC. Notably, patients presenting with both low GNRI and low Hb levels exhibit the most unfavorable prognosis. The implementation of a nutritional management strategy may improve the prognosis of VPC patients with a low H‐GNRI score.

## Author Contributions

**Jieping Ying:** data curation (equal), investigation (equal), software (equal), writing – original draft (equal). **Suwen Zhu:** investigation (equal), writing – review and editing (equal). **Yingchun Cheng:** resources (equal). **Bei Wang:** supervision (equal). **Yueqin Wang:** conceptualization (equal), funding acquisition (equal), methodology (equal).

## Ethics Statement

This study received approval from the Ethics Committee of The First People's Hospital of Yancheng in 2021 (approval number: 2024‐k‐171).

## Conflicts of Interest

The authors declare no conflicts of interest.

## Data Availability

The data used to support the findings of this study are included within the article.
